# mmWave-RM: A Respiration Monitoring and Pattern Classification System Based on mmWave Radar

**DOI:** 10.3390/s24134315

**Published:** 2024-07-02

**Authors:** Zhanjun Hao, Yue Wang, Fenfang Li, Guozhen Ding, Yifei Gao

**Affiliations:** 1 College of Computer Science and Engineering, Northwest Normal University, Lanzhou 730070, China; 2022222225@nwnu.edu.cn (Y.W.); lifenfang@nwnu.edu.cn (F.L.); 2022222269@nwnu.edu.cn (G.D.); 2022212162@nwnu.edu.cn (Y.G.); 2 Gansu Province Internet of Things Engineering Research Center, Lanzhou 730070, China

**Keywords:** classification, convolutional neural network, FMCW millimetre wave radar, K-nearest neighbours, respiration detection, support vector machine

## Abstract

Breathing is one of the body’s most basic functions and abnormal breathing can indicate underlying cardiopulmonary problems. Monitoring respiratory abnormalities can help with early detection and reduce the risk of cardiopulmonary diseases. In this study, a 77 GHz frequency-modulated continuous wave (FMCW) millimetre-wave (mmWave) radar was used to detect different types of respiratory signals from the human body in a non-contact manner for respiratory monitoring (RM). To solve the problem of noise interference in the daily environment on the recognition of different breathing patterns, the system utilised breathing signals captured by the millimetre-wave radar. Firstly, we filtered out most of the static noise using a signal superposition method and designed an elliptical filter to obtain a more accurate image of the breathing waveforms between 0.1 Hz and 0.5 Hz. Secondly, combined with the histogram of oriented gradient (HOG) feature extraction algorithm, K-nearest neighbours (KNN), convolutional neural network (CNN), and HOG support vector machine (G-SVM) were used to classify four breathing modes, namely, normal breathing, slow and deep breathing, quick breathing, and meningitic breathing. The overall accuracy reached up to 94.75%. Therefore, this study effectively supports daily medical monitoring.

## 1. Introduction

Respiration is an essential life-sustaining process, regulated by the exchange of external and internal respiration, as well as by neural and metabolic mechanisms for gas exchange and acid–base balance. The central nervous system controls human respiration [1]. Numerous factors influence respiration, including oxygen and carbon dioxide levels, acid–base balance, and emotional and physical activity. Understanding the background and fundamentals of respiration is essential for studying and monitoring respiration-related health states. The automatic regulation of respiratory rate and depth adjusts to the body’s needs and environment, with normal or abnormal breathing reflecting cardiorespiratory health and well-being. An abnormal respiratory state may indicate the presence of a disease [2]. When a person’s respiratory value significantly deviates from the normal range, there may be a potential respiratory disease. Therefore, daily monitoring of respiratory status provides valuable insights into a person’s health and well-being.

Respiratory diseases often manifest through abnormalities in respiratory depth, rate, and rhythm, creating a range of distinct respiratory states that can indicate illness [3]. However, in today’s work environment, a sedentary lifestyle has become the norm for many. Sitting limits the expansion of the chest and lungs, potentially leading to reduced lung function and an elevated risk of chronic respiratory conditions. Consequently, there is growing concern for the respiratory health of those working in offices. Traditional monitoring methods typically involve wearable devices, which can be uncomfortable for daily use and are often forgotten, thus compromising continuous health monitoring. In response to these challenges, researchers have begun exploring non-contact respiratory detection methods, such as wireless sensing. Among these, non-contact radar sensors stand out as they eliminate the need for physical contact with the subject, offering an enhanced user experience and enabling more flexible, round-the-clock human health monitoring. This approach has garnered increasing attention as a viable alternative to traditional methods [4].

Although using millimetre-wave radar for respiratory monitoring and classification has non-contact and high-resolution advantages, it still faces many challenges. Firstly, the respiratory signals from the millimetre-wave radar are susceptible to interference from environmental noise and multipath effects. Although existing studies have used filtering and signal-processing techniques to suppress noise, the effect is limited in the case of severe multipath interference [5]. Second, most studies use statistical features and traditional machine learning algorithms (e.g., support vector machine) for respiratory pattern classification, which perform poorly when dealing with complex respiratory patterns [6]. In 2022, He et al. used ultra-wideband (UWB) radar sensors for non-contact respiratory pattern recognition and developed a classification method to incorporate random forests, but the classification accuracy was insufficient [7]. In addition, most existing studies have focused on respiration monitoring under single conditions, with less discussion on factors such as different environments, distances, and angles. Alizadeh et al. used a millimetre-wave radar for respiration and heart rate detection. Although they validated the system’s effectiveness in static environments, there was a lack of evaluation of the system’s performance in complex environments [8]. These challenges limit the wide application of millimetre wave radar technology in respiratory monitoring and classification. To this end, this paper proposes an improved millimetre-wave radar respiration monitoring method that eliminates static noise through signal superposition, employs image processing techniques for respiration pattern classification, and systematically discusses the recognition effects in different environments, distances, and angles, thus effectively addressing the shortcomings of existing techniques. The main contributions of this study are summarised as follows:

(1) This paper uses multiple antenna superposition to suppress static noise, enhance the submerged vital sign signals, and achieve effective extraction of respiratory signals under different influencing factors.

(2) This paper systematically discusses the effects of different factors on monitoring different respiratory patterns and the extent to which they are affected by different distances and other factors.

(3) The mmWave-RM system developed in this paper captures human breathing non-contact and uses 2D images to classify different breathing patterns in a daily office environment. The G-SVM has proved to be effective after extensive experiments, with an accuracy of 94.75%.

## 2. Related Work

To evaluate the health status of the human respiratory system, the traditional method employed by doctors involves auscultating breath sounds [9]. These methods are inexpensive and easy to operate but suffer from subjective errors and tend to result in misdiagnosis, particularly when performed by inexperienced auscultators. In contrast, with the advancement of vital signal detection technology, the advent of devices such as cardiac tracers [10], electrocardiographs [11,12], and piezoresistive respiratory sensing systems with wearable housings for respiratory measurements [13,14] has provided more advanced tools for the medical field. With increasing public awareness of health, smart bracelets and watches on the market are garnering increasing attention. Including a respiratory health research function in the latest smart bracelet launched by HUAWEI highlights the growing emphasis companies place on respiratory status monitoring.

Existing health monitoring methods are primarily categorised into medical image and wearable device [15,16] monitoring. Medical imaging techniques, such as computed tomography (CT) scanning [17] and X-ray technology [18], provide high-resolution images, but they are expensive, bulky, and highly radioactive, making them unsuitable for daily personnel monitoring. These techniques are typically used after a person has developed symptoms of discomfort, lacking better predictability. Wearable devices generally require physical contact, may cause discomfort when worn for long periods, and are more limited in some scenarios.

The research trend in recent years has gradually shifted towards non-contact respiratory monitoring techniques. In non-contact monitoring, Wi-Fi sensing technology [19] is one of the main tools. Respiratory rate measurements can be successfully achieved via peak detection, channel state information (CSI) amplitude, CSI phase, and received signal strength (RSS). However, there are some limitations in their sensitivity and measurement accuracy. Kontou et al. [20] used an 80 MHz Wi-Fi device to collect fine-grained wireless channel state information (CSI) with a simple, shallow artificial neural network for respiratory frequency detection. Guo et al. [21] introduced BreatheBand, a fine-grained and robust respiratory monitoring system based on commercial Wi-Fi signals. Subcarrier selection and independent component analysis were used to extract respiratory components from raw CSI signals. However, CSI and RSS are not sensitive enough to detect subtle respiratory motion variations, and the measurement accuracy decreases significantly when the subject’s position is beyond a specified distance.

In contrast, radar technology performs much better in fine-grained sensing problems. FMCW radars [22,23] provide wider-range, higher-resolution detection than other radars through frequency modulation and can capture micromovements such as breathing [24] and heartbeat movements. Many current studies use high-frequency band FMCW millimetre wave radars [25] for vital signal detection. Therefore, this paper aims to design a system capable of classifying four respiratory patterns based on 77 GHz FMCW millimetre wave radar.

In previous studies, Miao et al. [26] developed an SVM-based classifier combining three features to classify four respiratory patterns with up to 93% accuracy in 2017. Feng et al. [27] developed a K-nearest neighbour (KNN)-based classifier using FPGA to implement six respiratory patterns for classification, achieving an overall accuracy of 73%. In the literature [28], CNN and SVM were combined to solve the classification problem of breath sounds, and the best classification accuracy reached up to 83%. In 2023, Hong et al. [29] proposed a 1D-SNN-based human breathing pattern detection model and a merged segmentation algorithm to classify multiple breathing patterns.

In this paper, based on FMCW radar combined with machine learning, the mmWave-RM system is proposed to classify human respiratory patterns, and the effects of multiple factors on respiratory patterns are considered. Unlike previous studies that used statistical features for classification [30], this system uses image processing to accurately classify four respiratory modes: normal breathing, slow and deep breathing, quick breathing, and meningitic breathing. The mmWave-RM system performs more accurately in respiratory modes classification, has a wide range of application prospects, and can be used for daily detection and medical assistance.

## 3. Proposed System

This study presents an overview of the general architecture of the mmWave-RM system and a detailed description of the system’s modules and their workflow.

### 3.1. Respiratory Signal Modelling

**Figure 1:**
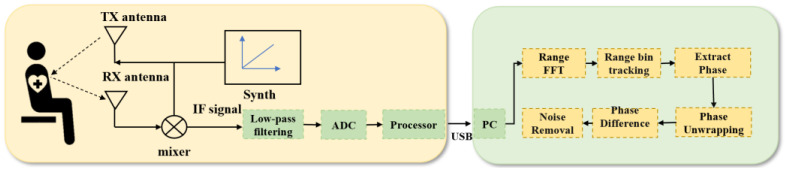
FMCW radar life detection signal principle and signal processing diagrams.

The basic principle of millimetre-wave radar detection of human respiration is through the signal sensing of small vibrations caused by chest undulation. The radar transmits a specific waveform of electromagnetic waves, which irradiates the movement of the chest wall. This generates echoes through the demodulation of the human chest wall after Doppler modulation of the echoes, to obtain information on thoracic cavity displacement containing respiratory and heartbeat parameters. The detection principle and the signal-processing process are shown in Figure 1.

The FMCW radar operates with the synthesiser, generating a linear FM pulse emitted by the transmitting antenna (TX antenna), called a Chirp. A Chirp is a single transmission, and the transmitting signal of the nth Chirp can be expressed as the following equation:

(1) $$F_{n}\left(t\right)={A_{T}exp\left\{-j\left(2\pi f_{c}t+\pi \frac{B}{T}t^{2}\right)\right\}}^{\;}$$

where $A_{T}$ is the amplitude of the transmit signal, $f_{c}$ is the transmit signal carrier, B is the transmit signal bandwidth, T is the transmit signal sweep time, and $\frac{B}{T}$ is the FM slope.

When the pulse reaches the object under test, the reflection of the linear FM pulse by the object generates a reflective FM pulse and is captured by the receiving antenna (RX antenna). The echo signal $R_{n}\left(t\right)$ can be expressed as the following equation:

(2) $$R_{n}\left(t\right)=A_{R}exp\left\{-j[(2\pi f_{c}(t-t_{d})+\pi \frac{B}{T}{\left(t-t_{d}\right)}^{2}]\right\}$$

where $A_{R}$ is the amplitude of the received signal, and $t_{d}$ is the delay time of the echo signal.

The mixer then combines the TX and RX signals to produce an intermediate frequency (IF) signal, expressed as follows:

(3) $${S_{IF}\left(t\right)=F}_{n}\left(t\right)\cdot R_{n}^{*}\left(t\right)\;=A_{T}A_{R}exp\left\{-j\left(2\pi f_{c}t+\pi \frac{B}{T}t^{2}\right)+j[(2\pi f_{c}(t-t_{d})+\pi \frac{B}{T}{\left(t-t_{d}\right)}^{2}]\right\}\;=Aexp\left\{-j\left(2\pi f_{c}t_{d}+\pi \frac{2B}{T}tt_{d}-\pi \frac{B}{T}{t_{d}}^{2}\right)\right\}$$

where A is the product of $A_{T}$ and $A_{R}$, and $R_{n}^{*}\left(t\right)$ is the complex conjugate of $R_{n}\left(t\right)$.

Respiratory motion modelling accurately describes the periodic motion of the human thoracic cavity, which is essential for extracting useful respiratory information from radar echo signals. Therefore, after obtaining the IF signal, the human chest displacement motion is modelled based on the characteristics of human respiration. The frequency of human respiration is between 0.1 Hz and 0.5 Hz, and the thoracic cavity expands and contracts rhythmically during respiration. The respiration motion model can be obtained by repeating this periodic cycle [31]:

(4) $$x_{r}(t)=A_{r}(0.5-{sin}^{p}\left(\pi f_{r}\left(t-\frac{\left\lfloor t\cdot f_{r}\right\rfloor }{f_{r}}\right)\right))\;0\leq \;t\leq 1/f_{r}$$

where t is the time, $f_{r}$ is the respiratory rate, the exponent p of the sin function controls the tip rounding as well as the overall shape, and ${1/f}_{r}$ is the repetition interval.

### 3.2. System Overview

**Figure 2:**
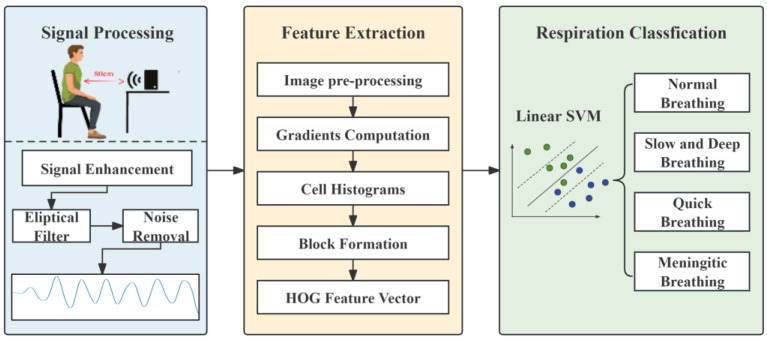
FMCW-RM system workflow.

This study focuses on the non-contact system mmWave-RM, which relies on FMCW millimetre-wave radar. The overall system flow is shown in Figure 2. The FMCW-RM system primarily comprises three modules: (1) The Signal Processing Module. This module handles the initial processing of respiratory data collected by IWR1843 millimetre-wave radar. It performs signal processing, including phase difference calculation and fixed band filtering, to obtain a respiratory signal. This ensures that the processed data truly reflects respiration. (2) The Feature Extraction Module. In this module, an algorithm based on HOG is utilised to extract features from the respiratory waveform image. (3) The Breathing pattern classification Module. This module collaborates with an SVM classifier to classify different breathing patterns.

### 3.3. Breathing Pattern Definition

**Figure 3:**
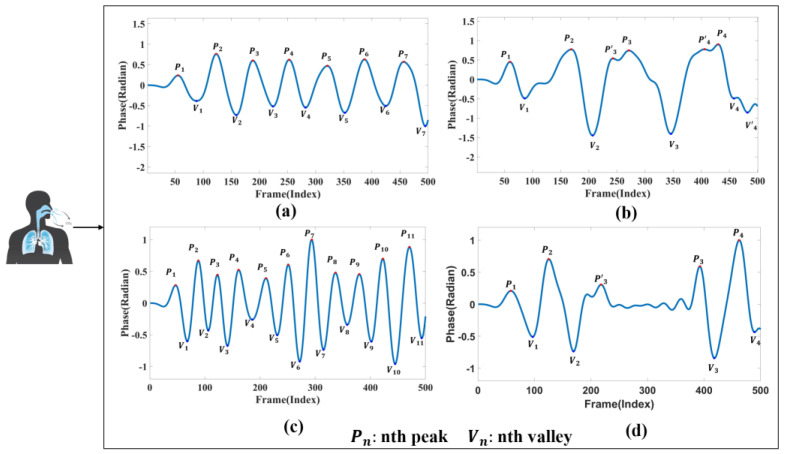
Respiratory time-domain waveforms. (**a**) Normal breathing. (**b**) Slow and deep breathing. (**c**) Quick breathing. (**d**) Meningitic breathing.

In this study, four distinct breathing patterns were detected: (a) normal breathing, (b) slow and deep breathing, (c) quick breathing, and (d) meningitic breathing. The time-domain waveforms of these breathing patterns are shown in Figure 3. As evident from Figure 3, there are notable differences in the waveforms of the four breathing patterns. The first pattern, normal breathing, exhibits a relatively stable waveform, as illustrated in Figure 3a. The second pattern, slow and deep breathing, is characterised by deeper breathing with more significant amplitude than the other breathing patterns, as shown in Figure 3b. The third pattern, quick breathing, features an intense respiratory waveform with a significantly increased number of breaths, as presented in Figure 3c. Finally, the fourth pattern, meningitic breathing, involves a breath-holding period under normal respiratory conditions followed by a return to normal breathing, as presented in Figure 3d.

The primary purpose of this study was to distinguish the disparities in respiratory signals among individuals under normal and abnormal physiological conditions. Therefore, respiratory patterns were categorised as either normal breathing or those associated with respiratory disease. As illustrated in Figure 3, the first category represents the respiratory state of an adult with normal breathing. Typically, the respiratory rate for adults in a resting state ranges from 12 to 20 breaths per minute [32,33]. The remaining three breathing patterns chosen for this study are modelled based on the characteristics of different respiratory disease symptoms.

The second type of breathing, slow and deep breathing, occurs during severe metabolic acidosis. When the extracellular fluid lacks bicarbonate, the pH value decreases, promoting the body to deepen respiration to discharge carbon dioxide and compensate for the extracellular acid–base imbalance. Common scenarios include diabetic ketoacidosis and uremic acidosis [34].

The third type of quick breathing, hyperventilation, is characterised by an elevated breathing rate. It often occurs in individuals with asthma, who may experience chest tightness, shortness of breath, and accelerated breathing during an attack. In mild cases, patients typically require regular medication. However, in severe cases, supervised treatment is necessary, as a sudden onset of breathlessness can be life-threatening.

The fourth type of meningitic breathing is characterised by alternating periods of respiration and apnoea. This phenomenon occurs due to decreased respiratory centre excitability or severe hypoxia, which prevents chemoreceptors from stimulating the normal concentration of carbon dioxide in the blood to excite the respiratory centre. Consequently, respiration gradually weakens until it stops temporarily, allowing the blood’s carbon dioxide concentration to accumulate during the apnoea period. This buildup stimulates the respiratory centre, initiating the next respiration cycle. This type of respiration is most prevalent in encephalitis and meningitis.

The respiratory states chosen for this study are intricately linked to prevalent respiratory ailments [35], and their differentiation not only facilitates daily surveillance of respiratory conditions but also helps patients identify the potential nature of their illness, thereby serving as a valuable tool for medical intervention.

### 3.4. Signal Processing Module

FMCW radar systems acquire the object’s distance, velocity, and angle under test by capturing the reflected signal. By analysing the IF signal $S_{IF}\left(t\right)$, the displacement information of the target can be further extracted. Firstly, the signals at different distances are grouped together by range bin to achieve accurate target positioning. Then, the target phase is obtained by performing FFT on the signal $S_{IF}\left(t\right)$.

The human respiration-induced thoracic displacement $x_{r}(t)$ is a periodic signal reflected in the phase change of the IF signal, and the displacement ∆d of the target induces a phase change in the FMCW signal, which is then the phase change between successive measurements:

(5) $$∆\emptyset_{b}=\frac{4\pi }{\lambda }∆d$$

where $∆\emptyset_{b}$ is the phase change of the beat signal, ∆d is the slight change in the thoracic cavity induced by the human body when breathing, and λ is the wavelength.

**Figure 4:**
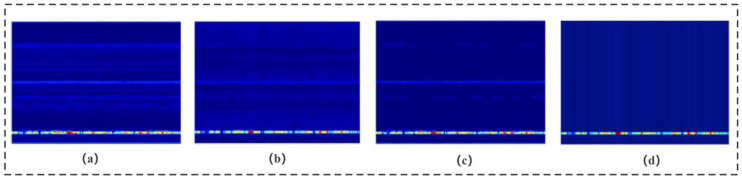
Comparison of de-noising methods. (**a**) Raw signal. (**b**) Average phase cancellation. (**c**) Moving target indication. (**d**) Signal overlay.

In millimetre-wave radar propagation, factors such as target distance, target absorption, and environmental interference can cause the life signal to be easily overwhelmed by noise. Figure 4 illustrates the effectiveness of three different denoising methods applied to the original signal. The average phase cancellation method partially attenuates the noise but leaves noticeable residual noise. The moving target indication method further reduces noise, yet fails to eliminate it completely. In contrast, the signal overlay method demonstrates superior denoising performance, nearly eliminating the noise and significantly enhancing the signal. Consequently, this module primarily employs the signal overlay method to effectively extract vital signals.

The data collected from multiple receiving antennas of the FMCW radar provide insights into the radar’s perception of human respiration at various time points [36]. By performing IQ complex summation and phase alignment using the cross-correlation function, we superimpose the signals from the four receiving antennas to achieve a more accurate and robust detection of vital signals.

**Figure 5:**
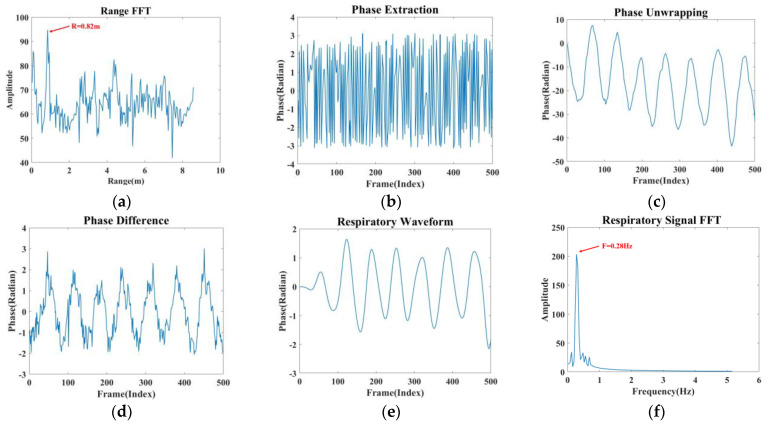
Signal processing. (**a**) Range FFT (**b**) Phase extraction. (**c**) Phase unwrapping. (**d**) Phase difference. (**e**) Respiratory signal. (**f**) Spectral estimation.

As illustrated in Figure 4, the static noise is effectively filtered following signal superposition, and the respiratory signal is also enhanced. The enhanced signal processing process is depicted in Figure 5, and the specific processing steps are as follows:

(1) A one-dimensional FFT is performed on the IF signal to determine the correct range bin, which aids in localising the human thoracic position, as shown in Figure 5a.

(2) The extracted phase information in Figure 5b is unwrapped, and phase expansion is achieved by subtracting 2π from the phase to produce Figure 5c.

(3) Taking d(t) as the unwrapped phase, the phase difference operation is performed on the unwound phase using the backward difference, i.e., $d\left(t\right)-d(t-1)$, which enhances the respiratory signal and removes the phase drift to produce Figure 5d.

(4) Since the frequency of human respiration is in the range of 0.1–0.5 Hz, this paper designs an elliptical filter according to the respiration frequency range, which only allows signals from 0.1 Hz to 0.5 Hz to pass through. The respiration signal can be obtained by smoothing and filtering the image after this step, as shown in Figure 5e.

(5) Figure 5f is obtained by performing a fast Fourier transform on the respiratory signal. The analysis revealed that the resulting waveform’s frequency was 0.28 Hz, which falls within the respiratory frequency range. The four modes’ processed respiratory waveforms serve as the module’s outputs.

### 3.5. Feature Extraction Module

**Figure 6:**
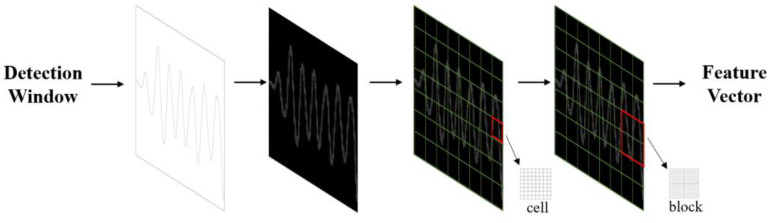
Flowchart of feature extraction.

After processing the data in Section 3.4, specific feature extraction algorithms were utilised to extract the characteristics of the respiratory waveform image. Abnormalities in frequency, depth, and rhythm often indicate respiratory disorders. As the human body experiences different respiratory states, the features of the respiratory signals may vary slightly. Therefore, selecting and extracting appropriate features is crucial for accurately assessing various respiratory conditions. Since the respiratory signals are enhanced in the signal processing module, more distinguishable differences can be observed in the extracted waveforms of different respiratory states. Therefore, this study adopted the image classification method to classify the waveforms of different respiratory modes. For feature extraction, this study chose the histogram of oriented gradient (HOG) algorithm that Navneet Dalal and Bill Triggs proposed in 2005 [37]. This algorithm offers significant advantages in image processing and effectively captures local texture and edge orientation information. It constructs features based on the gradient direction statistics in the image’s local regions, enhancing classification accuracy. The algorithm is insensitive to colour, illumination, and scale variations, and can extract valuable features from the waveform’s shape, texture, and gradient information. The overall flow of the HOG feature extraction algorithm is shown in Figure 6.

The detailed process of working on the feature extraction module is as follows:

Step 1: Pre-processing operations are performed on the images, which include four steps: image loading, resizing, greyscale, and data storage segmentation. (1) The image data is retrieved from the file system and diverse categories of images are stored in separate folders. (2) The images are resized using the resize function to ensure that each image has a size of 256 × 256 pixels. (3) The greyscale process transforms each pixel in the colour image from an RGB value to a greyscale value. (4) The processed images are segmented into four categories, normal breathing, slow and deep breathing, quick breathing, and meningitic breathing, which are then stored.

Step 2: The horizontal and vertical gradient values are calculated for each pixel and combined to calculate the total gradient value. The HOG within the rectangular region is utilised to obtain the desired feature description. The formulae for calculating the gradient and orientation are shown in (6) and (7):

(6) $$G\left(x,y\right)=\sqrt{{G_{x}}^{2}\left(x,y\right)+{G_{y}}^{2}\left(x,y\right)}$$

(7) $$\theta \left(x,y\right)=\tan^{-1}\frac{G_{y}\left(x,y\right)}{G_{x}\left(x,y\right)}$$

where $G_{x}\left(x,y\right)$ and $G_{y}\left(x,y\right)$ denote the gradient values of the image pixels in the x,y directions, $G\left(x,y\right)$ denotes the total gradient magnitude, and $\theta \left(x,y\right)$ denotes the gradient direction.

Step 3: The image is divided into cell-like units containing several pixels. Then, the gradient histogram of each cell-like unit is calculated, and the cellular units are projected onto the gradient direction based on their gradient direction and magnitude. The gradient direction range is divided into nine direction intervals, and the sum of the gradient magnitude within each interval is statistically calculated.

Step 4: Several adjacent cell-like units are combined into a cell block, and the gradient histogram of each cell block can be considered a feature vector. The feature vectors of all cell blocks are concatenated to obtain the final HOG feature vector.

Step 5: Using principal component analysis (PCA) for dimensionality reduction, all samples are centred by subtracting the mean $x_{i}\leftarrow x_{i}-\sum_{i=1}^{n}x_{i}$. Subsequently, the covariance matrix of the samples $xx^{T}$ is computed, and the eigenvalue decomposition is performed on the covariance matrix. The unit eigenvector corresponding to the largest m eigenvalues is taken, $\omega_{1,\;\;}{\;\omega }_{2,\;}\;\omega_{3,}\cdots \omega_{m}$. Finally, the projection matrix is obtained, which is the output of module II.

### 3.6. Breathing Pattern Classification Module

In this study, an SVM classifier was chosen to classify breathing patterns. SVM, as a supervised learning algorithm, aims to find a hyperplane that can optimally separate different classes as in Equation (8). In this module, we let the set of linearly separable samples be $\left(x_{1},y_{1}\right),\ldots ,\left(x_{l},y_{l}\right),x_{i}\in R^{n},y_{i}=1,-1,i=1,\ldots ,l$. Then, the hyperplane can be expressed as Equation (9) so that the positive and negative class inputs in the training samples are on both sides of this hyperplane. It is shown as follows:

(8) w×x−b=0

(9) $$\omega^{T}x+b=0$$

At this point, a parameter par (ω,b) exists, enabling $y_{i}=sgn\left(\omega^{T}x+b\right),i=1,\ldots ,l$, maximizing the interval between the two classes. Then, the problem of finding the optimal plane is transformed into the optimization problem defined by Equation (10).

(10) $$\left\{\begin{array}{c} min:\;J\left(w,b,a\right)=\frac{1}{2}\omega^{T}\omega -\sum_{i=1}^{N}a_{i}\left[y_{i}\left(\omega^{T}x_{i}+b\right)-1\right]①\\ y_{i}\left(\omega^{T}x_{i}+b\right)\geq +1\;\;\;② \end{array}\right.$$

where $a_{i}$ are the constrained Lagrange multipliers, and since they are all inequality constraints, these multipliers are all non-negative. The partial derivatives of Equation ① give Equation (11):

(11) $$Q\left(a\right)=\sum_{j=1}^{N}a_{i}-\frac{1}{2}\sum_{i=1}^{N}\sum_{j=1}^{N}a_{i}a_{j}y_{i}y_{j}(x_{i},x_{j})$$

This equation is referred to as the dual form of Equation ①. Meanwhile, the optimal solution to the optimization problem must satisfy $a_{i}y_{i}\left(\omega^{T}x_{i}+b\right)-1=0$. Moreover, any $a_{i}\neq 0$ can be found as b. Since $\sum_{i=1}^{N}a_{i}y_{i}$ = 0, it can be inferred that most of the $a_{i}$ are equal to 0. The samples corresponding to $a_{i}$ not equal to 0 are called support vectors. The model performs well on relatively small datasets and has an excellent ability to generalise. It excels at overcoming the challenges of machine learning on small samples, can handle high-dimensional data, and avoids the problems of structure selection and local minima in neural networks. The model is trained using reshaped image data as input features and corresponding category labels.

In this study, we employed the SVM method to classify different breathing patterns based on the feature extraction of the HOG algorithm, resulting in HOG-SVM (G-SVM). The model was trained for classification after preprocessing and feature extraction of the data. The classifier’s training was based on a fivefold cross-validation method, where all the breathing samples were divided into five groups, and the samples with different breathing states were labelled as four predefined types of normal breathing, slow and deep breathing, quick breathing, and meningitic breathing, respectively. Four groups were used for training and one was used for testing, resulting in an overall ratio of 4:1 for training to testing sets.

The FMCW-RM system pseudo-code is as Algorithm 1:
**Algorithm 1: Steps of FMCW-RM****Input:** Dataset (M_i_,N_i_)(i = 1,2,⋯⋯,n)
**Output:** Classification accuracy
  1. img=resize(img,(256,256))
  2. G_magnitude = sqrt(power(Gx, 2) + power(Gy, 2))//Calculate gradient value
  3. G_angle =arctan2(Gx, Gy)
  4.   bins=Get_bins(G_magnitude, G_angle, cell_size, bin_count)// Calculate the histogram of the gradient 
  5. **function** Block_Vector(bins, cell_x, cell_y, bin_count)
  6. **For** i in range(0, self.cell_x − 1):
  7.  **For** j in range(0, self.cell_y − 1):
  8.    magnitude =mag(feature)// calculates the magnitude of feature
  9.  **end for**
10.  **end for**
11. **return block_vector**

12. **end function**
13. clf = svm.SVC( )//model training
14. clf.fit(train_data, train_target)
15. pred = clf.predict(test_data)// model prediction
16. accuracy = calculate_accuracy(test_target, pred)
17. **return accuracy**


## 4. Experimentation and Evaluation

The breathing signal extraction methods proposed in this study were primarily validated across varying distances, clothing, angles, and environments, ensuring the extraction of accurate and reliable breathing waveforms for classification. Additionally, the overall performance of the FMCW-RM system was evaluated in this section by comparing the recognition rates of different classifiers on the four breathing patterns.

### 4.1. Experimental Parameters and Environment Settings

**Figure 7:**
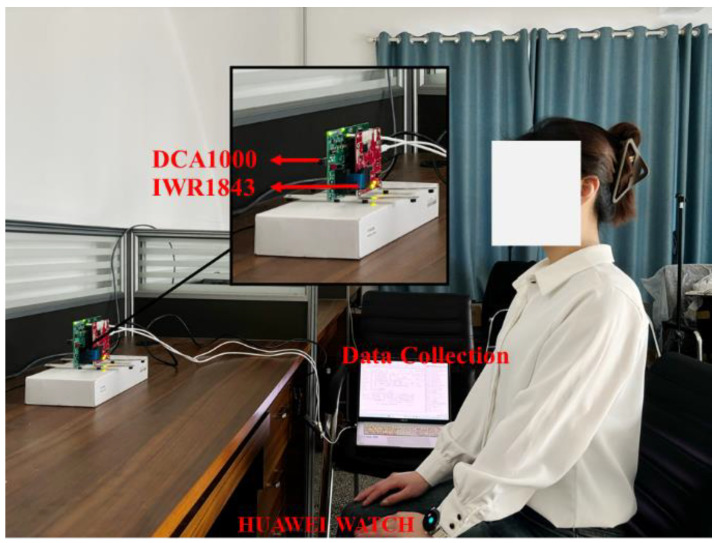
Experimental environment.

The equipment used in this study was the IWR1843BOOST radar and the DCA1000EVM data acquisition board, both from Texas Instruments, Dallas, TX, USA, for the acquisition of human vital signals. The IWR1843BOOST is a single-chip millimeter-wave radar sensor operating in the 76~81 GHz band with the parameter settings shown in Table 1. The radar featured three transmit antennas and four receive antennas, and the experimental setup is shown in Figure 7. For this experiment, the IWR1843BOOST millimetre-wave radar and the DCA1000EVM data acquisition board were positioned directly in front of the target to acquire the raw ADC data and transmit it to the computer via the USB data cable. When the computer received the raw data, the data was parsed and processed using MATLAB on a computer with an AMD Ryzen7 5800H processor and 16 G of RAM.

Data collection was performed in an office environment. The experimenter was seated in a chair at rest, with the radar positioned 0.8 m in front of the subject. The detectable area of the radar was aimed directly at the human chest at approximately 0.9 m. The acquired data were stored as a binary bin file. Table 2 shows the details of the subjects. In the experiment, the subjects needed to simulate three respiratory states, in addition to normal respiration. Each respiratory state needed to last for 25 s of collection time, and each respiratory mode collected 300 sets of data, of which 240 sets were used as training data and 60 sets were used as test data. Although the vital signals were easily drowned out by random body movements and external environmental noise, the denoising method used in this paper was more effective in suppressing static noise. Therefore, the subjects only needed to avoid significant limb movements during the experiment. All collected samples were sorted into four breathing states: normal breathing, slow and deep breathing, quick breathing, and meningitic breathing.

### 4.2. Reliability Validation of Millimetre Wave Radar Measurement Methods

To verify the reliability of the millimetre-wave radar respiration measurements, the respiration waveforms were used to estimate the respiration rate via peak detection, spectral estimation, and autocorrelation after the millimetre-wave radar had acquired the signals. In this section, all signal data was normalised so that the signal amplitude was between −1 and 1. As external influences and human respiration are unstable, we set the threshold for solving the counts to ±0.2 to allow for sufficient amplitude variation between respiration waveforms and to exclude inconspicuous peaks to mitigate over-detection problems. This process was discarded if the first waveform obtained was a trough. Secondly, if a peak remains a peak after one wave, it is not counted until the next trough is encountered. This trough, along with the previous peak, is considered as one full breath. As in Figure 3b, $P_{3}$, $V_{3}$ is a complete breath and, ${P_{3}}^{\prime }$ is discarded. The breathing training function of the HUAWEI WATCH GT2 also recorded the number of breaths during the experiment. The process confirmed the validity of the acquired signal by calculating the error between the measured respiratory rate and the number of gusts recorded. The total error was 0.5 times. The experimental data is shown in Table 3.

**Figure 8:**
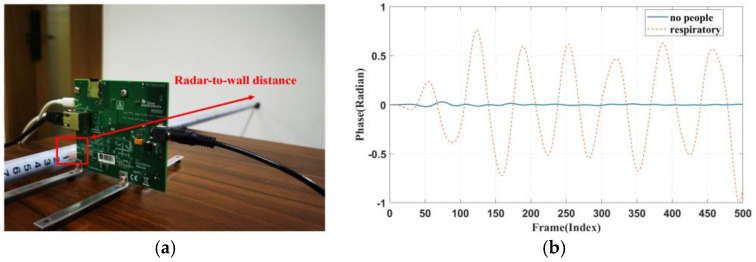
(**a**) Control setup. (**b**) Respiratory waveform obtained when facing the wall.

Furthermore, this study conducted a series of control experiments. In these experiments, the millimetre wave radar faced the wall at a distance of 0.8 m, and data was collected. The collected data was then processed and filtered between 0.1 Hz and 0.5 Hz. Figure 8 shows the control experiment scene and the waveforms obtained from the radar when placed on the chest cavity and when it faced the wall. The amplitude of the waveform when no breathing activity was present was close to zero, indicating that the signal detected in this frequency range was indeed a breathing signal.

### 4.3. Physical Environment Analysis

#### 4.3.1. Distance Analysis

**Figure 9:**
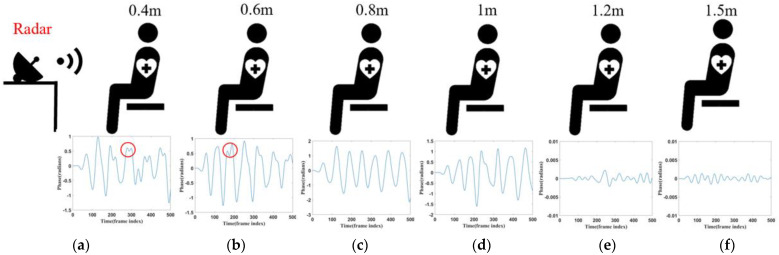
(**a**) Corresponding waveform of 0.4 m. (**b**) Corresponding waveform of 0.6 m. (**c**) Corresponding waveform of 0.8 m. (**d**) Corresponding waveform of 1 m. (**e**) Corresponding waveform of 1.2 m. (**f**) Corresponding waveform of 1.5 m.

This section examines the effectiveness of monitoring respiratory signals at different distances, with experiments conducted at six distance points. The distances between the radar and the subject’s chest were set at 0.4 m, 0.6 m, 0.8 m, 1 m, 1.2 m, and 1.5 m. At each distance, 20 datasets were collected. Each subject’s respiratory signals were recorded for 25 s, and the corresponding waveforms are shown in Figure 9. As shown in Figure 9, the respiratory waveforms of the subjects were extracted at six different distances. At the positions of 0.4 m and 0.6 m, due to their closer distance, the signal was enhanced, however, the noise was similarly enhanced, and thus the acquired image will be shown as a wave with two small spikes, as shown by the circled portion in Figure 9a,b. The signal was significantly weaker at a 1.2 m and 1.5 m distance. At a distance of 0.8 m and 1 m, the waveform effect was optimal, but at a distance of 0.8 m, when the signal amplitude was more significant, the signal was more stable. Therefore, based on a combination of five experimental objects of data, in most cases, the distance of 0.8 m yielded the best results, making 0.8 m the preferred experimental distance.

**Figure 10:**
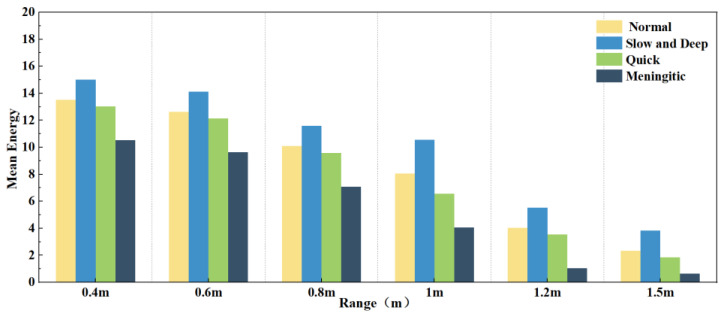
Average energy of different respiration patterns at different distances.

Figure 10 further analyses the effect of different distances on the four modes of normal, slow and deep, quick, and meningitic breathing, demonstrating the changes in the average energy of each breathing mode at different distances. It can be seen that the average energy of all breathing modes decreased significantly with increasing distance. However, the effects on different breathing modes were not the same. The energy was always the highest in slow and deep breathing since chest vibration is most pronounced in this group. Although the average energy of normal and slow and deep breathing decreased with distance, the magnitude of the energy decrease was similar, reflecting the small difference in the effect of distance on the two breathing modes. Intermittent pauses in breathing, on the other hand, showed the most pronounced decrease in energy, which could be attributed to the fact that the amplitude of breathing is much greater at closer distances than at farther distances, and that the breath-holding phase greatly reduces the overall energy of the signal.

#### 4.3.2. Analysis of Diversity in Personnel Status

The essence of radar-based vital signs extraction is detecting minute vibrations in the chest cavity. However, clothing worn by subjects can have an impact on the signals. To investigate the influence of clothing on the extraction of various breathing patterns, this study collected respiratory signals from five volunteers wearing three types of clothing, T-shirts, thin jackets, and coats, in an office environment.

**Figure 11:**
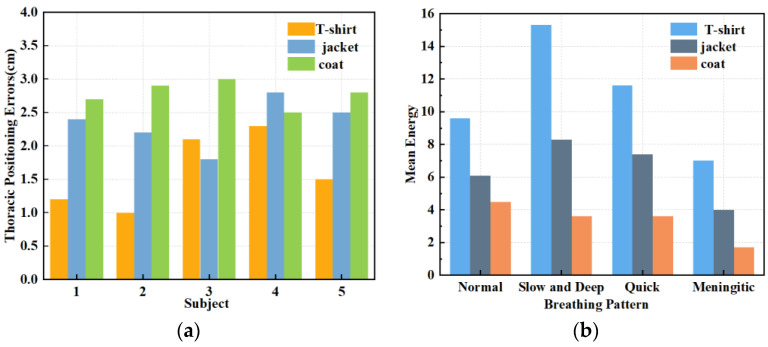
(**a**) Effect of different garment thicknesses on thoracic positioning. (**b**) Effect of varying garment thicknesses on different breathing patterns.

When subjects wear different types of clothing, the localisation of the chest during respiratory signal extraction can be inaccurate due to variations in thickness, potentially leading to signal distortion. Therefore, in this set of experiments, we evaluated three clothing types: a 2 mm thick T-shirt, a 4 mm thick jacket, and a 7 mm thick coat. Figure 11a shows the errors in chest localisation for different clothing thicknesses. Despite differences in thickness ranging from 2–5 mm, the error in chest positioning exceeded 1 cm. Figure 11b shows the effect of the three clothing thicknesses on the four breathing patterns: normal, slow and deep, quick, and meningitic. Notably, the instantaneous energy of slow and deep breathing was highest when wearing thinner clothing, as the chest vibrations are most pronounced during deep breathing. However, as the clothing thickness increased, slow and deep breathing energy decreased the most, although the energy of all other breathing modes also decreased. Thiscan be attributed to the thickened clothing absorbing some signals, leading to reduced instantaneous energy across all breathing patterns. Therefore, it can be concluded that clothing thickness has a notable impact on respiratory signals, particularly during deep breathing, emphasising the importance of the subject wearing thin clothing for accurate signal detection. In practice, external noise will drown out part of the signal, and a slight increase in the thickness of the clothes will affect the accuracy, so in this paper, we chose a jacket with moderate thickness for subsequent experiments. Exploring the detection of life signals in more complex environments will be a problem that subsequent research endeavours will need to overcome.

#### 4.3.3. Perspective Analysis

**Figure 12:**
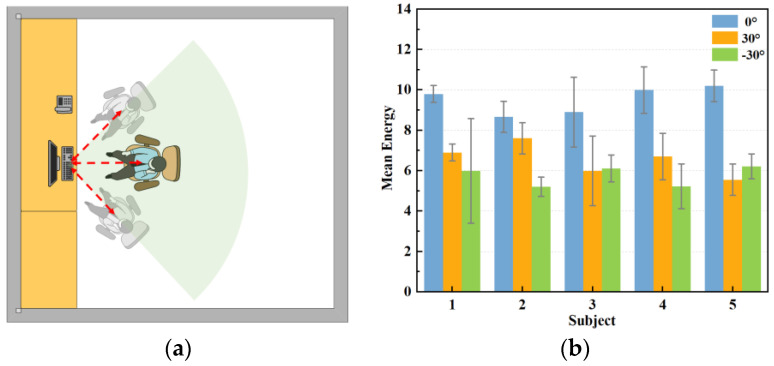
Measurement of respiratory signals at different angles. (**a**) Schematic diagram of measurement angles. (**b**) Respiratory energy at different angles.

To enhance the target detection accuracy and conserve energy, the antenna arrangement of the radar often assumes a fan-shaped configuration, resulting in a fan-shaped sensing area during data collection. This study aimed to monitor human respiration in an office setting when subjects simulate their typical sitting posture at work. We compared the orthogonal signals, where the radar was aimed at the centre of the human chest cavity, with signals at three angles, at zero degrees, thirty degrees to the left, and thirty degrees to the right. As Figure 12 demonstrates, 20 datasets were collected at each angle in this section to verify the extracted respiratory signals, yielding a total of 60 datasets.

**Figure 13:**
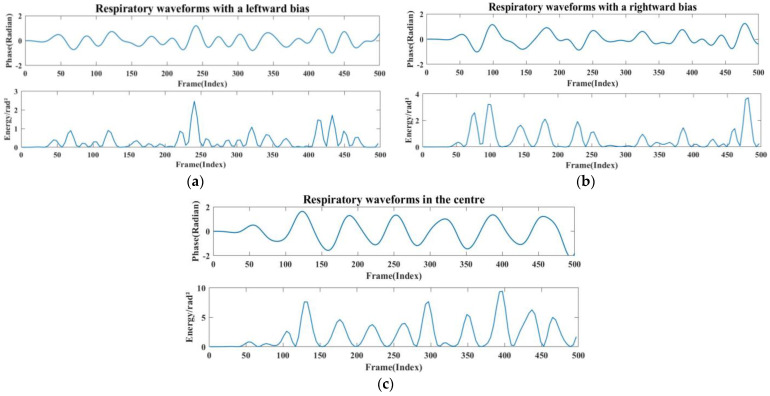
(**a**) Radar left-aligned to chest waveform and energy map. (**b**) Radar right-aligned to chest waveform and energy map. (**c**) Radar frontal to human chest waveform and energy map.

The stability of the positively aligned signals shows superior performance, as shown in Figure 12. This is because when the main flap of the radar and the human chest target are optimally aligned, the signal path is straighter, resulting in relatively uniform reflected signal intensity. Conversely, when the radar and the human chest are misaligned to the left or right, signal stability significantly diminishes, and signal amplitude also experiences a significant reduction. This occurs because the alignment between the radar’s main flap and the target is suboptimal, leading to increased signal dispersion and weakened reflection. In these scenarios, multipath effects and signal attenuation become more pronounced, further compromising signal stability. As shown in Figure 13, when the radar position is shifted by 30° to the left or right, the strength and energy of the respiratory signal are significantly reduced. Therefore, ensuring the stability of the radar’s alignment is critical for optimal signal quality in practical applications.

#### 4.3.4. Analysis of Different Experimental Environments

**Figure 14:**
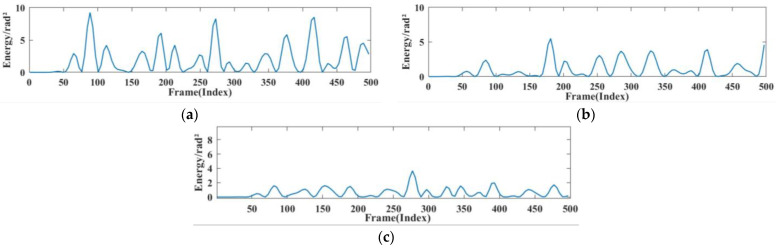
Breathing energy in different environments. (**a**) Quiet environment. (**b**) Working environment (**c**) noisy environment.

**Figure 15:**
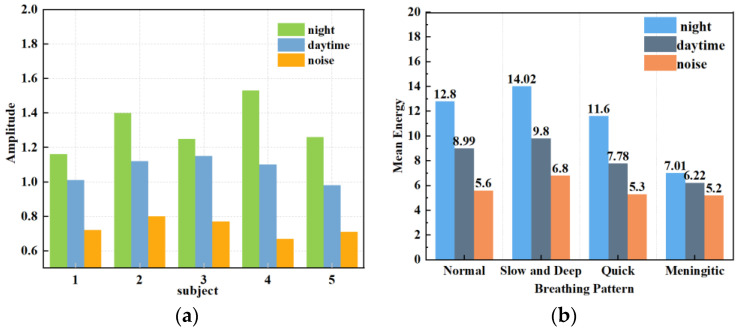
Respiratory amplitude in different environments. (**a**) Effects of different noise levels on respiratory monitoring of different subjects. (**b**) Effects of different noise levels on different breathing patterns.

The respiratory heartbeat serves as a micromotion signal if prone to being overwhelmed by noise, and both indoor and outdoor devices can cause the signal to undergo multiple reflections, ultimately interfering with the extraction of the respiratory signal. The indoor devices are stationary and invariant. In this section, we compared the extraction condition of the respiratory signal in a quiet nighttime environment, a typical daytime environment, and a noisy environment. In this case, the normal environment had people other than the subject sitting in the experimental environment but not in the radar monitoring range, to simulate a work scene with no other noise generation. The noisy environment incorporated mobile phones playing music and the sound of personnel talking. The experimental results are shown in Figure 14 and Figure 15.

Figure 14 shows the instantaneous energy variations of respiratory signals across different environments. In a quiet environment, the maximum energy of the respiratory signal ranged around 10; in a typical daytime working environment, the energy decreased to 5 to 7; and in a noisy environment, the energy decreased further. This indicates that as environmental noise increases, the energy of the respiratory signal decays rapidly, exhibiting various degrees of change. As shown in Figure 15a, the amplitude of human respiration was most pronounced in a quiet nighttime environment. The waveforms obtained in a daytime setting were slightly disturbed by noise, resulting in an average decrease of 0.248 in the amplitude of extracted respiration signals. On average, the amplitude decreased by 0.586 in noisy environments compared to that in quiet environments. Figure 15b reflects the amplitudes and energies of various breathing patterns across the three settings. It shows that meningitic breathing was least affected by changes in external ambient noise, presumably due to the breath-holding period inherent in this type of breathing pattern, causing respiratory signals to be less influenced by filtering out the high-frequency noise components. Respiratory signals obtained across various environments can count breaths, further validating the robustness of this study’s signal extraction and noise reduction techniques.

### 4.4. Classification Results

**Figure 16:**
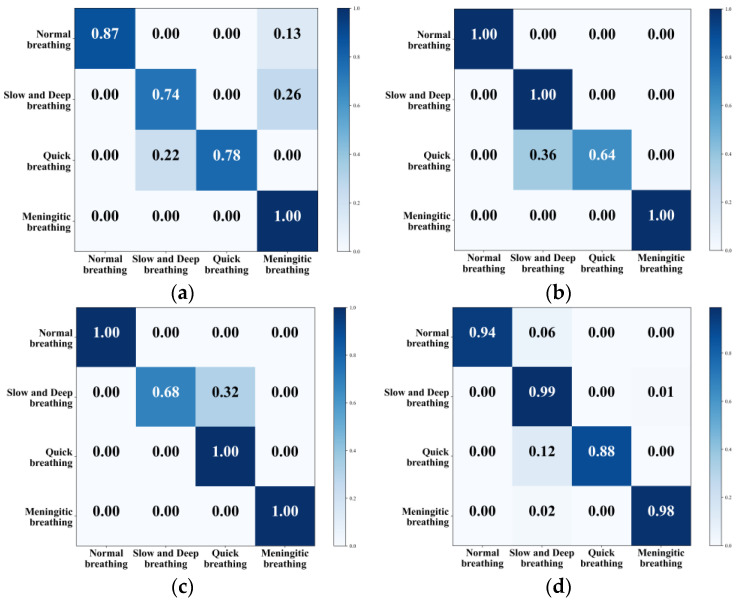
Confusion matrix for classification of different eigenvalues. (**a**) KNN. (**b**) SVM. (**c**) CNN+LSTM. (**d**) G-SVM.

This section classified four types of respiratory pattern data collected in the daily office environment. To comprehensively evaluate and quantify the model’s performance and visualise its classification effect, the confusion matrix was used to visualise the classification accuracy of different types of respiration, and the results are shown in Figure 16. Table 4 shows the accuracy of the model’s training and test sets using various features, which serve as metrics for evaluating the effectiveness and performance of the machine learning model.

All four methods can classify different kinds of respiratory states, with KNN achieving an overall classification accuracy of 84.75%. The model was more accurate in recognising meningitic respiration, while the recognition rate of the other three respiratory patterns was not very high. The overall classification accuracy of the traditional SVM was 91%, and this method had a high recognition rate for normal breathing, slow and deep breathing, and meningitic breathing, but only 64% for quick breathing. The mainstream CNN combined with LSTM had an overall recognition accuracy of 92%, but it was ineffective at recognising slow and deep breathing. To solve the problem of low recognition accuracy for both quick breathing and slow and deep breathing modes, this paper adopted the G-SVM method to extract features and classify them. This improved the classification accuracy of quick breathing and slow and deep breathing to 88% and 99% and improved the overall accuracy to 94.75%, enabling the classification of different respiratory states.

### 4.5. Comparison with Recent Research Work

We compared the method used by mmWave-RM with other recent research on respiratory pattern classification, as shown in Table 5. Both Mah et al. [15] and He et al. [7] used a random forest classifier to classify respiratory patterns. However, in [15] polynomial fitting was used for signal denoising, and respiratory depth was used as a feature, resulting in a classification accuracy of 87%. In [7], He et al. used singular value decomposition for signal denoising and combined it with time domain features for classification, which improved the accuracy. Purnomo et al. [38] processed signals using various techniques, extracted the MFCC features, and then classified them using the XGBoost model. Park et al. [16] proposed a new method based on the CNN model, which classified breathing patterns with an accuracy of more than 92%.

Considering the above result, compared to existing work, mmWave-RM employs a denoising technique based on signal superposition. This method can effectively improve signal quality and achieve accurate feature extraction, which, in combination with the classical SVM classifier, can achieve better classification of breathing patterns.

## 5. Conclusions

Respiratory status is a vital reference indicator reflecting human cardiopulmonary function. With the quick development of medical technology, more and more people are beginning to pay attention to monitoring their daily health status to achieve early prevention of respiratory diseases. In this study, FMCW millimetre-wave radar performs accurate extraction and classification of respiration signals based on the non-contact respiration-sensing method. This paper examined human respiration monitoring at different distances, different angles, different clothing thicknesses, and different environments, and classified four respiration modes in the daily office environment. The experiments were conducted to analyse the effectiveness of respiratory monitoring under different influencing factors and to classify different respiratory states using KNN, SVM, CNN, and G-SVM classifiers after processing the data collected in the office environment. The G-SVM classifier performed the best out of the four respiratory modes, with an overall accuracy rate of 94.75%. After many experiments, the effectiveness of using waveform images for classifying respiratory status was verified. However, the data acquisition process currently does not account for the impact of coughing and sneezing on respiration. Further research is needed to classify respiration states in more complex scenarios, which will also be our next step.

## Figures and Tables

**Table 1:** Radar parameter setting.

Parameters	Value
Start Frequency	77 GHZ
Bandwidth	4 GHZ
Number of Transmitting Antennas	1
Number of Receiving Antennas	4
Samples Per-Chirp	200
Chirp Duration	50 μs
Frame Duration	50 ms
Number of Chirps per Frame	2

**Table 2:** Detailed information on subjects.

Subject Number	Sex	Height (cm)	Weight (kg)
1	male	172	60
2	male	185	75
3	male	178	71
4	female	163	47
5	female	170	61

**Table 3:** A comparison of respiratory rates measured by millimeter-wave radar and HUAWEI WATCH GT2.

Number	Millimeter-Wave Radar (Breaths Times)	HUAWEI WATCH GT2 (Breaths Times)
1	10	9
2	7	7
3	8	8
4	8	7
5	7	8
6	9	8
7	10	9
8	7	7
9	11	10
10	9	8
Average	8.6	8.1

**Table 4:** Accuracy of different methods.

Method	Accuracy
KNN	84.75%
SVM	91%
CNN+LSTM	92%
G-SVM	94.75%

**Table 5:** Comparison of recent research work.

Method	Denoising Technology	Features	Model	Accuracy
[15]	Polynomial fit	Respiratory interval	Random Forest	87%
[7]	Differencing	MFCC	XGBoost	87%
[38]	Singular value decomposition	Time-domain features	Random Forest	90%
[16]	Not mentioned	Embedded features	CNN	92.34%
This Work	Signal overlay	HOG	SVM	94.75%

## Data Availability

Data are contained within the article.
